# Improving Nutritional and Functional Quality by Genome Editing of Crops: Status and Perspectives

**DOI:** 10.3389/fpls.2020.577313

**Published:** 2020-10-23

**Authors:** Hyung-Keun Ku, Sun-Hwa Ha

**Affiliations:** Department of Genetic Engineering and Graduate School of Biotechnology, College of Life Sciences, Kyung Hee University, Yongin, South Korea

**Keywords:** CRISPR, crop, genome editing, new breeding technology, quality improvement

## Abstract

Genome-editing tools including meganucleases, zinc finger nucleases, transcription activator-like effector nucleases and clustered regularly interspaced short palindromic repeats (CRISPR) system have been applied to improve the quality of staple, oilseed, and horticultural crops with great accuracy and efficiency compared to conventional breeding. In particular, the CRISPR method has proven to be a feasible, cost-effective and versatile tool allowing precise and efficient editing of plant genomes in recent years, showing great potential in crop improvement. Until now, various genome-edited crops with enhanced commercial value have been developed by not only global companies but also small laboratories in universities, suggesting low entry barriers with respect to manpower and capital. In this study, we review the current applications of genome editing technologies to improve the nutritional and functional quality and preferred traits of various crops. Combining this rapidly advancing genome-editing technology and conventional breeding will greatly extend the potential of genome-edited crops and their commercialization.

## Introduction

Crop improvement has continuously aimed to achieve higher yields, strong tolerance to biotic and abiotic stresses and enhanced nutritional quality. To achieve these, breeding techniques have been widely used to select desirable traits within crossbreeding species. Artificially inducing mutations using chemical or radioactive irradiation, in addition to naturally occurring mutations, have greatly expanded the diversity in selectable traits. However, these conventional methods are often labor-intensive and time-consuming and the rarity and randomness of significant mutations resulting in the emergence of desirable traits hinder the development of new commercial varieties (Pacher and Puchta, 2017). Since 1996, genetically modified (GM) crop varieties with traits of interest have been developed by introducing genes or genetic elements with known functions. Nevertheless, there are unproven concerns about potential health and environmental safety of GM crops developed by foreign gene introduction, requiring considerable time and financial investment to adhere to rigorous and excessive safety assessments (Prado et al., 2014).

With the potential to address these concerns, genome-editing tools have emerged as a new breeding technology and have been successfully used to modify crop genomes without traces of foreign gene introduction in a wide variety of plants. In a relatively short time, they have had a great influence on crop improvement, achieving higher accuracy and efficiency in genetic modification than conventional breeding (Chen et al., 2019). As a result, many genome-edited crops have been developed, and their numbers are increasing more rapidly compared to those developed by any other methods such as conventional breeding, natural and artificial mutant screening, and GM technology (Park et al., 2019).

The era of genome editing began with the use of several endonucleases to introduce site-specific DNA double-stranded breaks (DSBs) as toolboxes: zinc finger nucleases (ZFNs) (Kim et al., 1996), transcription activator-like effector nucleases (TALENs) (Christian et al., 2010), and clustered regularly interspaced short palindromic repeats (CRISPR)-associated 9 (Cas9) endonucleases (Jinek et al., 2012). After DSBs are generated using one of the above, the plant’s internal DNA repair systems can fix these DSBs either through imprecise non-homologous end joining, resulting in the insertion or deletion of nucleotides to induce gene knockouts, or through precise homologous recombination (HR) inducing gene replacements and insertions (Symington and Gautier, 2011). ZFNs and TALENs are produced by fusing the DNA cleavage domain of the endonuclease *Fok*I with the DNA-binding domains of the ZF or TALE proteins, which achieve sequence specificity via protein–DNA binding. Unlike ZFNs and TALENs, CRISPR/Cas9 relies on RNA–DNA binding guided by a 20-nt sequence in the single-guide RNA (sgRNA) in CRISPR and introduces DSBs through Cas9 (RNA-guided DNA endonuclease). The fact that addressability toward specific genomic DNA sequences using RNA-based CRISPR/Cas9 system is more convenient than protein-based methods, such as ZFNs and TALENs, has enabled the CRISPR/Cas9 method to be widely applied to develop the majority of genome-edited crops.

Recently, cases of crop improvement using genome-editing technologies have been rising exponentially. Among the target traits for crop improvement, nutritional and functional qualities are major objectives in the production of food, animal feed and raw materials. In this study, we review the recent developments of staple, oilseed and horticultural crops aiming to enhance diverse nutritional and functional quality traits with a focus primarily on commercial value (Table 1). Additionally, we summarize their current status and discuss the perspectives of genome editing technology for further quality improvement in these crops.

**TABLE 1:** Nutritional and functional quality-improved crops by gene-editing technologies.

**Crop class**	**Scientific name(Common name)**	**Target gene**	**Target function**	**Improved trait**	**Trait group**	**Editing method**	**Repair mechanism**	**Delivery technique**	**Reference**	**USDA’s answer to ‘Am I regulated?’/ Subject of commecialization**
**Staple crop**	*Oryza sativa* (Rice)	BADH2	Betaine aldehyde dehydrogenase	Increased fragrance content	Functional metabolite	TALEN	NHEJ	Agrobacterium-mediated transformation	Shan et al., 2015	
		SBEIIb	Starch branching enzyme	Increased amylose content	Starch	CRISPR/Cas9	NHEJ	Agrobacterium-mediated transformation	Sun et al., 2017	
		Waxy	Waxy protein/Granule-bound starch synthase	Improved glutinosity by lower amylose/amylopectin ratio	Starch	CRISPR/Cas9	NHEJ	Agrobacterium-mediated transformation	Zhang J. et al., 2018	
		rc	Rc/Basic helix–loop–helix transcription factor	Production of proanthocyanidins and anthocyanidins	Functional metabolite	CRISPR/Cas9	NHEJ	Agrobacterium-mediated transformation	Zhu et al., 2019	
	*Triticum aestivum* (Wheat)	α-Gliadin	α-Gliadin	Low-gluten wheat with strong reduction in α-gliadins	Protein	CRISPR/Cas9	HDR	Particle bombardment	Sánchez-León et al., 2018	
		GW2	Grain weight 2/RING-type E3 ubiquitin ligase	Increased grain weight and protein content	Protein	CRISPR/Cas9	NHEJ	Particle bombardment	Zhang Y. et al., 2018	
	*Zea mays* (Maize/Corn)	IPK1	Inositol-1,3,4,5,6-pentakisphosphate 2-kinase	Reduced phytic acid content	Anti-nutrient	ZFNs	HR	Whisker-mediated transformation	Shukla et al., 2009	Not considered to be regulated/Dow AgroScience 2012
		IPK1A, IPK, MRP4	Inositol phosphate kinase, Multidrug resistance-associated protein4	Reduced phytic acid content	Anti-nutrient	CRISPR/Cas9, TALEN	NHEJ	Agrobacterium-mediated transformation	Liang et al., 2014	
		MADS47	MADS-box protein zmmads47	Reduced zein protein	Protein	CRISPR/Cas9	NHEJ	Agrobacterium-mediated transformation	Qi et al., 2016	
		Wx1	Waxy protein/Granule-bound starch synthase	High-amylopectin starch	Starch	CRISPR/Cas9	NHEJ	Biolistic transformation	Waltz, 2016a	Not considered to be regulated/Dupont Pioneer 2016
	*Solanum tuberosum* (Potato)	GBSS	Granule-bound starch synthase	High-amylopectin starch	Starch	CRISPR/Cas9	NHEJ	PEG-mediated transfection	Andersson et al., 2017	
		GBSS	Granule bound starch synthase	Absence of amylose	Starch	CRISPR/Cas9	NHEJ	Ribonucleoprotein delivery	Andersson et al., 2018	
		GBSS1	Granule bound starch synthase I	Low amlyose starch in tubers	Starch	CRISPR/Cas9	NHEJ	Agrobacterium-mediated transformation	Kusano et al., 2018	
		PPO	Polyphenol oxidase	Reduced browning	Market value	TALEN	NHEJ	PEG-mediated transfection	–	Not considered to be regulated/Calyxt Inc.*, 2016
		PPO5	Polyphenol oxidase	Reduced black spot, enzymatic darkening and discoloration in potato tubers	Market value	TALEN	NHEJ	Agrobacterium-mediated transformation	–	Not considered to be regulated/Simplot Plant Sciences, 2016
		PPO2	Polyphenol oxidase	Reduced browning	Market value	CRISPR/Cas9	NHEJ	Ribonucleoprotein delivery	Gonzalez et al., 2020	
		16DOX	Steroid 16α-hydroxylase	Absence of steroidal glycoalkaloids	Anti-nutrient	CRISPR/Cas9	NHEJ	Agrobacterium rhizogenes	Nakayasu et al., 2018	
		SSR2	Sterol side chain reductase 2	Reduced steroidal glycoalkaloids	Anti-nutrient	TALEN	NHEJ	Agrobacterium-mediated transformation	Yasumoto et al., 2019	
		VInv	Vacuolar invertase	Reduced sugars in tubers causing reduced acrylamide in chips with improved cold storage and processing quality	Toxic substance	TALEN	NHEJ	PEG-mediated transfection	Clasen et al., 2016	Not considered to be regulated/Calyxt Inc.*, 2014
**Oilseed crop**	*Brassica napus* (Rapeseed)	FAD2_Aa	Fatty acid desaturase 2	Increased oleic acid content	Lipid	CRISPR/Cas9	NHEJ	Agrobacterium-mediated transformation	Okuzaki et al., 2018	
	*Camelina sativa* (Camelina)	DGAT1, PDAT1	Diacylglycerol acyltransferase, Phospholipid: diacylglycerol acyltransferase	Altered fatty acid composition with reduced oil content	Lipid	CRISPR/Cas9	NHEJ	Agrobacterium-mediated transformation	Aznar-Moreno and Durrett, 2017	
		FAD2	Delta-12 desaturase	Reduced levels of polyunsaturated fatty acids	Lipid	CRISPR/Cas9	NHEJ	Agrobacterium-mediated transformation	Morineau et al., 2017	
		FAD2	Fatty acid desaturase 2	Reduced levels of polyunsaturated fatty acids	Lipid	CRISPR/Cas9	NHEJ	Floral dip method	Jiang et al., 2017	
		FAE1	Fatty acid elongase 1	Reduced very long-chain fatty acids and increased C18 unsaturated fatty acids	Lipid	CRISPR/Cas9	NHEJ	Agrobacterium-mediated transformation	Ozseyhan et al., 2018	
	*Glycine max* (Soybean)	FAD2-1A, FAD2-1B	Fatty acid desaturase 2	Reduced levels of polyunsaturated fatty acids	Lipid	TALEN	NHEJ	Agrobacterium-mediated transformation	Haun et al., 2014	Approved/Calyxt Inc.*, 2019
		FAD2-1A, FAD2-1B, FAD3A	Fatty acid desaturase 2-1A and -1B,Fatty acid desaturase 3	Reduced levels of polyunsaturated fatty acids	Lipid	TALEN	NHEJ	Agrobacterium mediated transformation, Biolistic mediated transformation	Demorest et al., 2016	Not considered to be regulated/Calyxt Inc.*, 2015
**Horticulturural crop**	*Agaricus bisporus* (Mushroom)	PPO	Polyphenol oxidase	Reduced browning	Market value	CRISPR/Cas9	NHEJ	PEG-mediated transformation	Waltz, 2016b	Not considered to be regulated/Pennsylvania State University, 2016
	*Nicotiana tabacum* (Tobacco)	BBL	Berberine bridge enzyme-like	Reduced levels of nicotine	Toxic substance	Meganuclease	NHEJ	Agrobacterium-mediated transformation	–	Not considered to be regulated/North Carolina State University, 2017
	*Solanum lycopersicum* (Tomato)	ALC	Alcobaca (ALC)/NAC domain protein (NAC-NOR)	Delayed fruit ripening causing long-shelf life	Market value	CRISPR/Cas9	HDR	Agrobacterium-mediated transformation	Yu et al., 2017	
		ANT1	Anthocyanin mutant 1/Myb transcription factor	Increased contents of anthocyanin	Functional metabolite	TALEN, CRISPR/Cas9	HR	Agrobacterium-mediated transformation	Čermák et al., 2015	
		GAD2, GAD3	Glutamate decarboxylase 2 and 3	Higher γ-aminobutyric acid content	Functional metabolite	CRISPR/Cas9	NHEJ	Agrobacterium-mediated transformation	Nonaka et al., 2017	
		GABA-TP1, GABA-TP3, CAT9, SSADH	Pyruvate-dependent GABA transaminases 1 and 3, Cationic amino acid transporter, Succinate semialdehyde dehydrogenase	Higher γ-aminobutyric acid content	Functional metabolite	CRISPR/Cas9	NHEJ	Agrobacterium-mediated transformation	Li et al., 2018b	
		lncRNA1459	Ripening-related long non-coding RNA1459	Repressed fruit ripening, ethylene production and lycopene accumulation	Market value	CRISPR/Cas9	NHEJ	Agrobacterium-mediated transformation	Li et al., 2018a	
		L1L4/NF-YB6	LEAFY-COTYLEDON1-LIKE4/Nuclear transcription factor Y (NF-Y) gene	Reduced contents of the anti-nutrient oxalic acid	Anti-nutrient	ZFN	NHEJ	Electroporation	Gago et al., 2017	
		MYB12	R2R3-MYB transcription factor	Pink colortrait by absence of yellow-colored flavonoid naringenin chalcone in tomato peels	Market value	CRISPR/Cas9	NHEJ	Agrobacterium-mediated transformation	Deng et al., 2018	
		RIN	MADS-box transcription factor/Ripening inhibitor	Delayed fruit ripening	Market value	CRISPR/Cas9	NHEJ	Agrobacterium-mediated transformation	Ito et al., 2015	
		RIN/LeMADS	MADS-box transcription factor/Ripening inhibitor	Delayed fruit ripening and reduced ethylene production	Market value	CRISPR/Cas9	NHEJ	Agrobacterium-mediated transformation	Jung et al., 2018	
		SGR1, LCY-E, Blc, LCY-B2	Stay-green 1, Lycopene ε-cyclase, Beta-lycopene cyclase, Lycopene β-cyclase 2	Increased contents of lycopene and beta-carotenoids	Functional metabolite	CRISPR/Cas9	NHEJ	Agrobacterium-mediated transformation	Li et al., 2018d	
	*Solanum pimpinellifolium* (Current tomato)	CycB, SP, O, MULT, FAS, FW2.2	Lycopene b-cyclase, Self-pruning, Ovate, Multiflora, Fasciated, Fruit weight 2.2	Increased contents of lycopene (5x) as nutritional value with alteration in the size (3x) and number (10x) of the fruits	Functional metabolite	CRISPR/Cas9	NHEJ	Agrobacterium-mediated transformation	Zsögön et al., 2018	
		GGP1, SP, SP5, CLV3, WUS	GGP1 encoding a vitamin C–biosynthetic enzyme, Self-pruning, Self-pruning 5g, CLV3, Homeobox-encoding gene WUS	Increased contents of vitamin C	Functional metabolite	CRISPR/Cas9	NHEJ	Agrobacterium-mediated transformation	Li et al., 2018c	
	*Vitis vinifera* (Grape)	IdnDH	L-idonate dehydrogenase	Reduced levels of tartaric acid	Anti-nutrient	CRISPR/Cas9	NHEJ	Agrobacterium-mediated transformation	Ren et al., 2016	

* Calyxt Inc. was renamed from Cellectis Plant Sciences.

## Staple Crops

To sustain human life, food security is crucial to provide food for the rapidly growing world population and cope with increasing meat consumption. Staple crops are central to satisfying such high demand and improving crop quality in terms of nutritional value. Because of this, genome editing is actively used in staple crop agriculture (i.e., maize, wheat, rice, and potato).

Maize (*Zea mays*), is the world’s most cultivated cereal crop. It has a wide range of uses: from animal feed to biofuel and other nutritional products for human consumption, such as corn starch and corn syrup. When ingested by animals and humans, phytate, also called inositol 1,2,3,4,5,6-hexakisphosphate, plays as an anti-nutrient to reduce protein and mineral absorption by forming insoluble complexes with them (Feil, 2001). To reduce phytate content in corn, the *IPK1* gene encoding for inositol-1,3,4,5,6-pentakisphosphate 2-kinase was blocked using ZFNs (Shukla et al., 2009). Similar approaches have been tried using CRISPR/Cas9 and TALENs to knock-out *IPK1A, IPK*, and *MRP4*, which encode for two inositol phosphate kinases and multidrug resistance-associated protein 4, respectively, in the phytate biosynthetic pathway (Liang et al., 2014). Zeins, the major corn storage proteins, are deficient in lysine and tryptophan (essential amino acids), contributing to corn’s poor nutritional quality (Geraghty et al., 1981). Previously, an *opaque2* (*o2*) mutant, which has defect in a corresponding gene encoding a maize endosperm-specific basic leucine zipper transcription factor, was identified as a low zein mutant among several maize starchy endosperm mutants; however, this mutant showed decreased yield (Hunter et al., 2002). To overcome the problem of reduced zein protein contents, RNA interference (RNAi) and CRISPR/Cas9 commonly targeted the gene *ZmMADS47* encoding a MADS-box protein, which is an interacting partner of O2 to activate zein gene promoter (Qi et al., 2016). Similar levels of reduction in zein content were reported, i.e., 16.8 and 12.5% decreases, in the kernels of *ZmMADS47 RNAi* and MADS/CAS9-21 lines, respectively (Qi et al., 2016; Qiao et al., 2016). They did not reach to a 64.6% decrease of zein content in the kernels of *o2* mutant yet. Another important target trait is starch (a polymeric carbohydrate consisting of amylose and amylopectin); its metabolism was altered using CRIPSR/Cas9 to disrupt the waxy gene (*Wx1*), which encodes a granule-bound starch synthase (GBSS) being responsible for synthesizing amylose in maize. Several versions of waxy mutants have been exclusively developed to contain only branched amylopectin, which is a commodity in processed foods, adhesives and high-gloss paper. To further improve agronomic traits on the purpose of commercialization, the CRISPR-editing of *Wx1* was applied to an elite commercial variety and crossbred as CRISPR-waxy hybrids (Waltz, 2016a). They were presented as the first CRISPR-edited crops available for cultivation and selling that were not subject to regulation by the United States Department of Agriculture (USDA).

Wheat (*Triticum aestivum*) was the first crop cultivated by humans according to the archeological record and is currently the second most grown cereal crop globally. However, the wheat storage protein, gluten, can trigger some health issues in some individuals, such as celiac and non-celiac disease and gluten ataxia (Sapone et al., 2011). To reduce the contents of α-gliadin in the gluten protein, knockouts of α-gliadin genes were generated using CRISPR/Cas9, and their immunoreactivity was reduced by 85%. Through further confirmation of transgene-free and no off-target mutations, this CRISPR-hypoimmunogenic wheat was found to have low gluten content and was therefore, possibly suitable for the production of low-gluten foodstuffs and as a source line for introgression into elite wheat varieties (Sánchez-León et al., 2018). CRISPR methods have also been applied to enhance total wheat protein content with increased grain weight by knocking out the *GW2* gene, which encodes a RING-type E3 ubiquitin ligase known to regulate the cell number of spikelet hulls (Su et al., 2011; Zhang Y. et al., 2018).

Rice (*Oryza sativa*) grains make up 20% of the world’s dietary energy supply and more than three billion people across the globe consume rice daily (Birla et al., 2017). The first quality improvements made through genome editing for this crop aimed to produce fragrances (Shan et al., 2015). Special fragrant rice varieties like the Indian Basmati and the Thai Jasmine types are popular among consumers and have a higher market price than common rice. To create this fragrance from common rice, the TALEN technique was used to disrupt the *BADH2* gene encoding for betaine aldehyde dehydrogenase (BADH), which produces a competing compound, γ-aminobutyric acid (GABA), instead of the main flavor compound, 2-acetyl-1-pyrroline (2-AP), from the same primary substrate of γ-aminobutyraldehyde (Shan et al., 2015). Like in other cereal crops, rice starch has a large influence on glutinosity for cooking and eating quality (Juliano, 1998). By knocking out the starch branching enzyme gene, *SBEIIb*, using CRISPR/Cas9, a high-amylose and low-viscosity rice variety was produced (Sun et al., 2017). Conversely, a low-amylose and glutinous rice was produced by knocking out the GBSS gene, *Waxy*, using CRISPR/Cas9 (Zhang J. et al., 2018). A rice cultivar with white pericarp (caused by the recessive *rc* allele due to a 14-bp frameshift deletion in the seventh exon of the *Rc* gene) was recently genome-edited using CRISPR-Cas9 to produce a phenotype with a red pericarp, which is functionally restored just like the dominant *Rc* allele, through an additional deletion that results in-frame mutations (Zhu et al., 2019).

Potato (*Solanum tuberosum*) is the world’s fourth-largest staple food crop after maize, wheat and rice. As an important tuberous crop, it plays a key role in providing an adequate nutritional balance, especially in developing countries. Its importance in global food supply has been achieved by refrigeration that extends postharvest shelf life by inhibiting sprouting during storage. However, this cold treatment can also stimulate the accumulation of reducing sugars (e.g., glucose and fructose) that cause elevated levels of a potential carcinogen compounds (acrylamide) by reacting with free amino acids when processed under high-temperatures (i.e., during cooking) (Tareke et al., 2002). To avoid this, the *VInv* gene encoding for vacuolar invertase, which catalyzes the breakdown of sucrose into glucose and fructose, was knocked-out using TALEN (Clasen et al., 2016). This *VInv*-knockout potato was commercialized by Cellectis Plant Sciences (now Calyxt Inc.) with undetectable levels of reducing sugars in tubers and reduced levels of acrylamide in processed products. Additionally, potato tubers accumulate steroidal glycoalkaloids (SGAs) α-solanine and α-chaconine that confer a bitter taste and exhibit toxicity against various organisms (Friedman, 2006). For this reason, reducing the SGA tuber content has been a long-term goal in potato breeding programs. To achieve this goal, TALEN and CRISPR/Cas9 were used to knockout the SSR2 gene encoding for sterol side chain reductase 2 and the *St16DOX* gene encoding for the steroid 16α-hydroxylase in the SGA biosynthetic pathway. This method prevented SGA accumulation in potato tuber and hairy roots, respectively (Nakayasu et al., 2018; Yasumoto et al., 2019). Meanwhile, high amylopectin (amylose-free) starch has been an important common trait in staple crops due its commercial value in the food and manufacturing paper industries. In potato starchy tubers, the GBSS gene was successfully knocked-out to generate high-amylopectin potato using three different CRISPR/Cas9 methods: PEG-mediated transfection, ribonucleoproteins (RNPs) and *Agrobacterium*-mediated transformation, respectively (Andersson et al., 2017, 2018; Kusano et al., 2018). Another common target in several crops is polyphenol oxidase (PPO) that catalyzes the conversion of phenols to quinones, discoloring compounds which cause a devaluation of the crops and their processed products (Stevens and Davelaar, 1997). TALEN methods designed to knock out one of the PPO genes were used to decrease black spots/browning in potato tubers due to this compound. Commercialization progressed using different delivery techniques (PEG-mediated transfection or *Agrobacterium*-mediated transformation) by two companies (Calyxt Inc., and Simplot Plant Sciences). Currently, a *PPO*-knockout potato is in field trials with another *VInv*-knockout potato. This is because USDA cleared these genotypes for commercialization in 2016 without performing a regulatory process. More recently, CRISPR/Cas9 methods using RNPs as a delivery system were reported to develop potato varieties with reduced enzymatic browning in tubers (Gonzalez et al., 2020).

## Oilseed Crops

To obtain edible or industrial oils, humans have relied on oilseed crops such as soybean, rapeseed, sunflower, cotton and nuts, which are grown for oil extraction purposes. Crop breeders and biotechnologists have tried to modify the fatty acid profiles of their seeds to improve oil quality according to the desired purpose. Through transgenic approaches, seed oils of soybean and rapeseed have been successfully modified in the degree of saturation of fatty acids either by knocking down or by knocking out the corresponding desaturase genes via targeted RNA interference. Genome editing seems a good alternative to RNAi technology when no pleiotropic effects are caused by knocking out the corresponding gene in whole plant cells. However, RNAi is still preferable when tissue- and condition-specific expression is required because it can controlled by the choice of the promoter used. At present, the most preferred target of genome editing is the production of monounsaturated fatty acids, such as oleic acid (18:1), which is generally considered healthier and oxidatively more stable, and hence is associated with a longer shelf life than products containing polyunsaturated fatty acids (PUFAs) such as linoleic acid (18:2) (Clemente and Cahoon, 2009). For this reason, the fatty acid desaturase 2 gene (*FAD2*) encoding for delta-12 desaturase, which converts oleic acid to linoleic acid, was targeted and disrupted to alter the fatty acid profile by increasing the oleic to linoleic acid ratio in soybean, rapeseed, and camelina.

Soybean (*Glycine max*) is one of the world’s most widely used edible oil crops, with a global oil production estimated at 57 million t (28% of world supply) for 2019/2020 (USDA-FAS, 2020). Soybean oil quality was first improved using TALENs to carry targeted mutations in two *FAD2* genes (*FAD2-1A* and *FAD2-1B*), resulting in increased oleic acid levels (from 20 to 80%) and decreased linoleic acid levels (from 50 to 4.7%) (Haun et al., 2014). To further decrease the levels of linoleic acid (to 2.5%), Calyxt Inc., knocked-out the fatty acid desaturase 3 gene (*FAD3*) encoding for delta-15 desaturase by directly delivering TALENs into previous *fad2-1a fad2-1b* soybean plants (Haun et al., 2014; Demorest et al., 2016). The latter was first commercialized for sale as a High-Oleic Soybean Oil in the United States market (Calyxt, 2019).

Camelina (*Camelina sativa*) has been given remarkable attention over the past decade, as its oil traits are distinct from those of other oilseed crops with high levels of omega-3 lipids and tocopherols, and it is easy to genetically engineer it (Haun et al., 2014; Demorest et al., 2016; Faure and Tepfer, 2016). The first target of genome editing in camelina was *FAD2*, which was independently knocked-out using CRISPR/Cas9 by two research goups in 2017 (Jiang et al., 2017; Morineau et al., 2017). As *C. sativa* is a hexaploid species; these reports described the use of gRNA to simultaneously target all three *FAD2* loci, with the common results showing a significant increase in oleic acid and a decrease in the levels of PUFAs, such as linoleic acid and linolenic acid. The efficiency of the CRISPR/Cas method in knocking out all homeologs of target genes in polyploid species was again confirmed using a carefully designed gRNA identical to *CsDGAT1* or *CsPDAT1* encoding diacylglycerol acyltransferase or phospholipid:diacylglycerol acyltransferase in all three camelina subgenomes, which are involved in triacylglycerol synthesis (Aznar-Moreno and Durrett, 2017). Meanwhile, camelina seed oil contains a significant amount of very long-chain fatty acids (VLCFAs) that may be undesirable for industrial or consumptive use. To reduce the levels of VLCFAs, the *FAE1* gene, which encodes for fatty acid elongase1, was deactivated by CRISPR/Cas9, resulting in increased levels of C18 unsaturated fatty acids (including oleic, linoleic, and α-linolenic acids) and reduced levels of C20–C24 VLCFAs (including eicosenoic acid and erucic acid) to less than 2% of the total fatty acid content, compared to over 22% in the wild type (Ozseyhan et al., 2018).

Commonly targeted in crops such as soybean and camelina, the *FAD2* gene was also targeted in rapeseed (*Brassica napus*) (Okuzaki et al., 2018). As the complete inactivation of all *FAD2* genes is known to affect plant development (e.g., twisted leaves and delayed bolting in camelina), a single mutant of *FAD2_Aa* edited with CRISPR/Cas9 led to slightly higher contents of oleic acid and lower contents of linoleic acid in rapeseed, which highlights the potential use of the remaining two *FAD2* genes (*FAD2_Ca* and *FAD2Cb*) as a target to further increase oleic acid content in rapeseed, without any harmful effects (Okuzaki et al., 2018).

## Horticultural Crops

Horticultural crops include vegetables and fruits that are important sources of carbohydrates, proteins, organic acids, vitamins, and minerals. However, these crops tend to be quickly perishable and may continuously ripen due to respiration after harvest. Crop breeders are thus seeking to further enhance their nutritional functionality, extend storage duration, and improve processing suitability in terms of color, texture, size, and flavor to achieve higher market value.

The most important horticultural crop requiring such quality improvement is tomato (*Solanum lycopersicum*) due to its economic and nutritional importance. To date, tomato crops are some of the most genome-edited crops. Genome targeting for HR in tomato has seen early successes compared to other crops, achieving precise genome modifications with similar efficiencies using both TALENs- and CRISPR/Cas9-based gene editing with geminivirus replicons as an exogenously supplied DNA donor template (Čermák et al., 2015). At the time, the target trait of interest was color selection, which is determined by anthocyanin production. Knocking-in of the cauliflower mosaic virus 35S promoter at the upstream of the anthocyanin mutant 1 gene (*ANT1*, Myb transcription factor) in the tomato genome resulted in purple tomatoes with high anthocyanin content. Meanwhile, plant breeders have focused their efforts on delaying fruit ripening to extend tomato storage duration and shelf life. A ripening inhibitor (*RIN*) gene, which encodes for the MADS-box transcriptional factor, was disrupted using CRISPR/Cas9 in different cultivars of Ailsa Craig, Mamirio, and Golden Bell varieties (Ito et al., 2015; Jung et al., 2018). All *RIN*-defective tomato mutants exhibited delayed ripening of fruits with less red pigmentation due to decreased lycopene production. Another attempt to delay ripening using CRISPR/Cas9 targeted long non-coding RNA1459 (*lncRNA1459*) (Li et al., 2018a); the edited mutants showed repressed ethylene production and lycopene accumulation, as well as delayed ripening. Another approach using homology-directed repair (HDR) to achieve a longer shelf life through delayed ripening targeted the *ALC* (Alcobaca) gene, which has a non-synonymous mutation in the allele of the non-ripening (*Nor*) gene (Yu et al., 2017). CRISPR/Cas9 introduced the *alc* gene, which contained only one single-base substitution (thymine by adenine), as a replacement via the HDR repair pathway using a DNA donor template, which was inserted into the vector downstream of sgRNA. Gene-edited *alc* homozygous tomatoes had excellent storage performance, and homozygous recessive breeding elites also showed a long shelf life.

Tomato quality improvement has also aimed to enhance the accumulation of functional metabolites. For instance, compared to other major horticultural crops, tomatoes contain relatively high levels of GABA, a metabolite that prevents hypertension when consumed daily (Matsumoto et al., 1997). A C-terminal autoinhibitory domain of *SlGAD2* and *SlGAD3* encoding glutamate decarboxylase (GAD), a key enzyme in GABA biosynthesis, negatively regulates the enzymatic function of GAD activity. To increase GABA content in tomato, this domain was deleted using CRISPR/Cas9. Introducing a stop codon immediately before the autoinhibitory domain increased GABA accumulation by a factor of 15 (Nonaka et al., 2017). Another GABA-enhanced tomato was generated using multiplexed CRISPR/Cas9, during which five key genes encoding for pyruvate-dependent GABA transaminase (GABA-TP 1, 2, and 3), cationic amino acid transporter (CAT9) and succinate semialdehyde dehydrogenase (SSADH) were targeted (Li et al., 2018b). A 19-fold increase in GABA content in tomato leaves resulted from these quadruple mutants with edited genes in *GABA-TP1*, *GABA-TP3*, *CAT9*, and *SSADH*. Tomato also produces high amounts of oxalic acid, an anti-nutrient metabolite that may inhibit the correct absorption of calcium due to the formation of insoluble salts (Gago et al., 2017). Among independent mutants, fruits with a targeted disruption of the *LEAFYCOTYLEDON1-LIKE4* (*L1L4*) gene, a nuclear transcription factor Y (NF-Y) known to regulate the biosynthesis of seed storage proteins and fatty acids, using ZFNs delivered by electroporation, several showed low oxalic acid levels, suggesting that the *L1L4* gene could regulate the accumulation of this compound and other metabolites such as citric acid, fructose, β-carotene, polyphenols, and antioxidants (Gago et al., 2017). Generally, tomato peel color depends on the amounts of naringenin chalcone (NarCh), a yellow-colored flavonoid. The absence of NarCh causes tomatoes to display a pink color in recessive yellow (*y*) mutants, the corresponding gene was identified as *SlMYB12*, which encodes an R2R3-MYB transcription factor (Ballester et al., 2010). The targeted disruption of *SlMYB12* using CRISPR/Cas9 successfully developed pink-fruited tomatoes, which are more popular than red-fruited tomato in Asian countries (Deng et al., 2018). Other target metabolites in tomato quality improvement include carotenoids, such as lycopene. To enrich lycopene in tomato, multiplexed genome editing using the CRISPR/Cas9 system was performed to target for five genes encoding for stay-green 1 (SGR1), lycopene ε-cyclase (LCY-E), beta-lycopene cyclase (Blc), lycopene β-cyclase 1 (LCY-B1), and lycopene β-cyclase 2 (LCY-B2) (Li et al., 2018d). Interestingly, the lycopene content in tomatoes subjected to genome editing was 5.1-fold higher when the *SGR1* was targeted alone, indicating that the effect of *SGR1* on the regulation of lycopene content was more pronounced than those of the other four cyclase genes for carotenoid biosynthesis (Li et al., 2018d).

Currant tomato (*Solanum pimpinellifolium*) is commonly grown in gardens but is still considered a wild species. This species exhibits remarkable stress tolerance and disease resistance, its *de novo* domestication is therefore promising for future commercial cultivation. To do this, six loci involved in fruit productivity, *SELF-PRUNING* (*SP* for general plant growth habit), *OVATE* (*O* for fruit shape), *MULTIFLORA* (*MULT* for fruit number), *FASCIATED* and *FRUIT WEIGHT 2.2* (*FAS* and *FW2.2* for fruit size), *LYCOPENE BETA-CYCLASE* (*CycB* for functional quality), were individually knocked-out using CRISPR/Cas9 (Zsögön et al., 2018). This altered fruit morphology, leading to increased fruit size (three-fold) and yield (10-fold). Furthermore, the fruit’s nutritional value was enhanced in terms of lycopene content (five-fold). Another approach to commercially cultivate currant tomatoes targeted five gene loci, including *SELF-PRUNING*, *SELF-PRUNING 5G*, *CLAVATA3*, *WUSCHEL*, and a vitamin C biosynthetic enzyme gene (*SlGGP1*) (Li et al., 2018c). To increase ascorbic acid levels, the upstream open reading frame (uORF) of *SlGGP1* was targeted using CRISPR/Cas9e. Edited currant tomatoes had higher foliar ascorbic acid content, demonstrating that uORF is a potentially suitable target for genome editing.

Other genome-edited horticultural crops have been reported in mushroom, tobacco, and grapes. As for potato tubers, postharvest browning (the oxidation of phenols to melanin initiated by PPO) occurs rapidly in mushrooms, resulting in decreased commercial value. To prevent browning, scientists used CRISP/Cas9 to knock-out one of six *PPO* genes in the common white button mushroom (*Agaricus bisporus*), by deleting just a few base pairs in the genome, which reduced enzymatic activity by 30% (Waltz, 2016b). Meantime, a low nicotine tobacco (*Nicotiana tabacum*) was developed by targeting members of the *berberine bridge enzyme-like* genes, which encode for enzymes that catalyze one of the final steps in nicotine biosynthesis, using a custom-designed meganuclease (USDA-APHIS, 2020). Genome-edited tobacco plants carried small deletion mutations (from 1 to 106 bp) and/or insertions (maximum size of 47 bp) at the targeted loci, thereby reducing the capacity of tobacco plants to produce nicotine. Grape (*Vitis vinifera*) synthesizes tartaric acid (TA) and accumulates it in significant quantities in the fruit. TA is one of three primary acids found in wine grapes, alongside malic and citric acid, which play a significant role in the development of wine flavor. To apply targeted genome editing in grapes, the *IdnDH* gene, which encodes for L-idonate dehydrogenase and regulates TA biosynthesis, was knocked-out using CRISPR-Cas9 (Ren et al., 2016). TA content was reduced by up to 36% in genome-edited grapes relative to control grapes, suggesting this method is an efficient and sufficiently specific tool for precise genome editing in this crop.

## Current Status for the Quality Improvement of Crops by Genome Editing

In general, the genome editing of crops is developing much more quickly than that of animals for many reasons, including the ease of selection on a large population and fewer bioethical concerns. As summarized in Table 1, many nutritional and functional quality traits have been modified and improved by the successful application of genome editing technologies in diverse crop plants. However, at present, we are at an early stage, with CRISPR-*wx1* maize, TALEN-*ppo* potato, and TALEN-*fad2* soybean yet to be successfully commercialized.

To accelerate their prompt and reasonable commercialization, technical limitations resulting from policy and intellectual property rights concerns should be resolved as a priority, and editing-associated technologies must continue to evolve. First, country-specific regulation policies should be carefully considered. Currently, Argentina, Brazil, Chile, Colombia, Japan, the United States, and Israel have established policies stating that genome-edited products are not required to follow GMO regulation if the final products do not contain foreign DNA sequences (Park et al., 2019). On the other hand, Europe and New Zealand upheld the legal ruling that genome-edited products should be regulated in the same way as GMO crops (Park et al., 2019). Nevertheless, the situation is progressing toward a set of policies to exclude some genome-edited crops from GMO regulation in many countries. For instance, the United States has a program named “Am I Regulated?” directed by the United States Department of Agriculture (USDA). This program, which belongs to the work performed by the Biotechnology Risk Analysis and Regulatory Operations Programs that oversees genetically engineered (GE) products, assesses whether products developed by GE technology in the United States meet the national regulations (USDA-APHIS, 2020). Several genome-edited crops have not been considered to be regulated in the United States through this process (Table 1). These included browning-reduced mushroom and nicotine-reduced tobacco cases that were developed by university laboratories rather than by companies, indicating that genome-editing technologies have generally low entry barriers with respect to manpower and capital. Moreover, universities can operate more freely. Globally, the high-oleic soybean variety developed by Calyxt Inc., was first approved in 2019 for commercialization, setting a precedent for the future licensing and commercialization for other genome-edited crops (Calyxt, 2019). At present, the main patent owners for genome editing technology, the Broad Institute and the University of California at Berkeley, have granted exclusive rights to surrogate companies, which major agricultural companies including Monsanto/Bayer Crop Science, Corteva^TM^, and Calyxt have acquired licenses (Ferreira et al., 2018). The license fees for intellectual property could restrict the ability of small- and medium-sized enterprises to commercialize their genome-edited crops. Therefore, to further advance the field of genome editing research for crop commercialization, it is suggested that reasonable licensing fees be imposed (Friedrichs et al., 2019).

The development of other technologies related to gene editing would also facilitate faster commercialization of genome-edited crops. In terms of integrating foreign DNAs, genome-edited crops have fewer potential risks compared to GM crops as most of the consequences of genome editing cause a change in only a small number of nucleotides, resulting in small genome changes that do not significantly differ from those found due to spontaneous mutations (Voytas and Gao, 2014). This should be positively reflected in legal policy such that genome-edited crops, compared to GMO, are comparatively free from regulation because exogenous DNA fragments could be carefully eliminated after the editing procedure either by the segregation of T-DNA when transgene methods are used or by the use of advanced transgene-free technologies, such as *in vitro* transcripts or RNPs. Recently, *in vitro* delivery methods of gene-editing materials have been successfully used in diverse plant systems and are continuously developing (Woo et al., 2015; Malnoy et al., 2016; Subburaj et al., 2016; Svitashev et al., 2016; Andersson et al., 2018; Liang et al., 2018). If no exogenous DNA traces can be completely confirmed, neither method can distinguish genome-edited crops from spontaneously-mutated crops. Therefore, the confirmation that genome-edited crops are transgene-free and without off-target mutations requires extensive genotyping during the early screening process and remains challenging. Recent technical advancements suggest diverse solutions to identify transgene-free genome-edited plants; these include fluorescence marker-assisted selection (Yu and Zhao, 2019), active interference element-mediated selection (Lu H.P. et al., 2017), programmed self-elimination systems (He et al., 2018), bolting-time assays (Cheng et al., 2019), and H_2_O_2_-based leaf assays (Wu et al., 2019).

Genome editing crops may also be improved by considering the target traits how RNAi technology has been used in the past, as well as the suitability of the host crop variety to be engineered. Considering the obvious difference between RNAi and genome editing, indiscreet genome editing can result in unexpected negative effects on agronomic traits by leading to pleiotropic effects in all parts of the crop plant. For example, a triple knockout of *FAD2* genes in camelina showed the highest oleic scid content but also resulted in drastic developmental defects, including slow growth, twisted leaves and delayed bolting traits. This was in stark contrast to the results of double-knockout camelina, which showed normal developmental traits (Morineau et al., 2017). In case of reduced-browning potato, one out of six genes associated with the PPO enzyme, which functions to protect the plants from disease, was eliminated; however, there is a concern that this present a potential risk against disease. After crops have been genome-edited, additional breeding processes require considerable time and capital to produce commercial varieties. To overcome these challenges, haploid induction editing technology enables direct editing of elite inbred lines by a single cross in nascent seeds of diverse monocot and dicot species, simplifying breeding processes, such as introgression, into elite commercial germplasm (Kelliher et al., 2019).

## Future Perspectives for Crop Quality Improvement by Genome Editing

Methodologically, genome-editing technologies have been continuously and rapidly advancing in diverse systems, including microorganisms and animals as well as plants. CRISPR/Cas9 has evolved to being able to perform small IN/DEL mutations at target sequences as well as precise single-base editing. Base editors usually consist of a sgRNA-guided Cas9 nickase (nCas9) fused with a deaminase that causes C to T/A to G base conversions (Komor et al., 2016; Gaudelli et al., 2017). These resources greatly increase the versatility of genome-editing tools, enabling the induction of precise mutations in crop genomes (Mishra et al., 2020). The other endonuclease (Cpf1) and newly discovered or designed Cas9 variants can recognize various protospacer adjacent motif (PAM) sequences, thereby resolving PAM-restricted site-specific cleavages (Hu et al., 2018; Manghwar et al., 2019). In particular, the delivery of Cpf1 proteins with *in vitro* transcribed or chemically synthesized target-specific gRNAs was found to successfully induce mutations at target sites in soybean and tobacco protoplasts, thereby proving the effectiveness of the Cpf1–gRNA complex as a DNA-free genome-editing method (Kim et al., 2017). The pace of application of these advanced technologies to crops will likely determine the speed and diversity of their use in practice.

**FIGURE 1:**
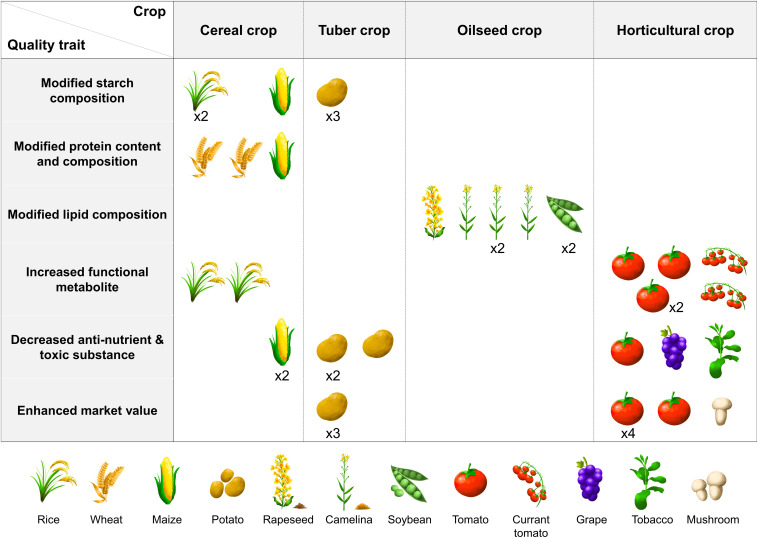
Preference of nutritional and functional quality traits in crops. The same trait in the same crop is denoted as a multiplied number.

Interestingly, different crops have shown different responses to genome editing, exhibiting their key characteristics (Figure 1). In addition, genome editing relies on the limited number of target traits suitable for improvement by editing. To diversify the nutritional and functional quality traits of crop plants for genome editing, more intensive discovery of target genes is required to investigate the association between genes and their controlled traits in metabolic pathways. To improve metabolic traits without introducing additional pleiotropic defects in crop plants, desirable targets for genome editing must be found from recessive traits among conventional genetic mutants in traditional breeding programs. For instance, fragrant, *waxy* and *rc* rice and *alc* tomato mutants have been recently used to develop genome-edited crops through the functional recovery of recessive alleles (Shan et al., 2015; Yu et al., 2017; Zhang J. et al., 2018; Zhu et al., 2019). At present, most research aiming at the nutritional and functional enhancement of crop species is at a stage where breeders are elucidating the function and regulatory mechanisms of various nutritional and functional metabolic pathways. To explore novel genes suitable for the development of crop varieties with improved target traits, the screening of large-scale mutant libraries constructed using CRISPR may be a good solution. These efforts on a whole-genome scale have been reported in rice (Lu Y. et al., 2017; Meng et al., 2017). The identification of significant mutations in gene-coding regions, the *cis* and *trans*-regulatory regions, and microRNAs would expand the range of possibilities for genome editing by providing an increased range of phenotypic variation available for crop improvement (Gallego-Bartolome et al., 2018; Zhang H. et al., 2018).

To date, the potential of genome-editing technology has been realized by the engineering of desirable traits in various crop plants with high efficiency and precision. The current state of this technology enables many applications suitable for improving plant traits, including nutritional and functional qualities. Continuous technological improvement and gaining better knowledge about the function of unknown genes will facilitate the future development of new genome-edited crops and their commercialization.

## Author Contributions

H-KK drafted the manuscript. S-HH envisaged and wrote the manuscript with H-KK. All authors reviewed and approved the final manuscript.

## Conflict of Interest

The authors declare that the research was conducted in the absence of any commercial or financial relationships that could be construed as a potential conflict of interest.
